# Synthesis and Structure of Oxygen Deficient Lead-Technetium Pyrochlore, the First Example of a Valence V Technetium Oxide

**DOI:** 10.3389/fchem.2021.706269

**Published:** 2021-07-01

**Authors:** Brendan J. Kennedy, Timothy A. Ablott, Maxim Avdeev, Melody L. Carter, Linda Losurdo, Matilde Saura-Muzquiz, Kevin J. Thorogood, Jimmy Ting, Kia S. Wallwork, Zhaoming Zhang, Hanliang Zhu, Gordon J. Thorogood

**Affiliations:** ^1^ School of Chemistry, The University of Sydney, Sydney, NSW, Australia; ^2^ Nuclear Fuel Cycle, Australian Nuclear Science and Technology Organisation, Kirrawee DC, NSW, Australia; ^3^ Australian Centre for Neutron Scattering, Australian Nuclear Science and Technology Organisation, Kirrawee DC, NSW, Australia; ^4^ Nuclear Materials, Australian Nuclear Science and Technology Organisation, Kirrawee DC, NSW, Australia; ^5^ Australian Synchrotron, Australian Nuclear Science and Technology Organisation, Clayton, VIC, Australia; ^6^ Department of Nuclear System Safety Engineering, Nagaoka University of Technology, Nagaoka, Japan

**Keywords:** technetium, pyrochlore, disorder, crystallography, spectroscopy

## Abstract

The structure of lead-technetium pyrochlore has been refined in space group $Fd\bar{3}m$ with *a* = 10.36584(2) Å using a combination of synchrotron X-ray and neutron powder diffraction data and confirmed via Electron Diffraction. The oxide is found to be oxygen deficient with a stoichiometry of Pb_2_Tc_2_O_7-d_. Displacive disorder of the Pb cations is evident from the refinements, as has been observed in Bi_2_Tc_2_O_7-d_. X-ray absorption spectroscopic measurements at the Tc K-edge demonstrate the valence of the Tc is greater than 4.0 as anticipated from the refined oxygen stoichiometry. Raman spectroscopy confirms the presence of disorder leading us to conclude that this pyrochlore is the first example of a valence V technetium oxide.

## Introduction

Technetium is unique amongst the transition metals in that no stable isotope exists. Technetium-99 is a major fission product of uranium-235 and is found in radioactive waste from nuclear fuel and, in certain jurisdictions, from the production of plutonium. Due to its long half-life (t_1/2_ = 2.1 × 10^5^ y) and high mobility through geological formations, the migration of ^99^Tc is a significant challenge in nuclear waste management and it is a major contributor to the amount of radiation in the biosphere. Other isotopes of Tc are also of considerable importance, for example ^99m^Tc is the most commonly employed isotope in nuclear medicine (Jurisson et al., 1993) and ^95m^Tc is used as a radioisotope tracer (Conversi, 1985).

In comparison to the extensive literature on the nuclear medical applications of soluble technetium species, there is a dearth of information on the solid-state chemistry of Tc. Indeed, the work by Muller, White and Roy (Muller et al., 1964) in 1964 remains one of the most comprehensive reported studies in this area. Over the ensuring decades a small number of experimental studies have appeared including our studies of some Tc perovskites (Avdeev et al., 2011; Rodriguez et al., 2011; Thorogood et al., 2011a; Mravlje et al., 2012; Reynolds et al., 2017a), TcO_2_ (Rodriguez et al., 2007; Reynolds et al., 2017b; Childs et al., 2018), inverse spinel (Thorogood et al., 2011b) and *A*TcO_4_ scheelites (Kennedy et al., 2019) together with the study of some Bi-Tc oxides by Rodriguez and co-workers (Rodriguez et al., 2008) and lanthanoid pyrochlores by Hartman et al. (2011). Theoretical studies of the lanthanoid technetate pyrochlores have also been reported (Weck et al., 2010). The extraordinary magnetic properties of SrTcO_3_ and CaTcO_3_ have ignited interest in Tc oxides.

Rodriguez et al*.* have reported the synthesis and crystal structure of the bismuth technetium pyrochlore Bi_2_Tc_2_O_7-d_ d ∼ 0.14 (Rodriguez et al., 2008). As observed for a number of other Bi pyrochlores, including Bi_2_Ru_2_O_7-d_ (Avdeev et al., 2002), this structure is characterised by static disorder of the bismuth cations, an effect that is enhanced by the 6s (Conversi, 1985) lone pair electrons. Muller and co-workers (Muller et al., 1964) reported that the ternary lead-technetium oxide also forms a pyrochlore structure, which they suggested may be non-stoichiometric. Beyerlein and co-workers (Beyerlein et al., 1984) reported that the analogous lead-ruthenium pyrochlore is non-stoichiometric and displays oxygen vacancy ordering, the stoichiometry actually being Pb_2_Ru_2_O_6.5_ and the resulting space group being $F\bar{4}3m$, rather than $Fd\bar{3}m$ as seen in Bi_2_Ru_2_O_7-d_ (Beyerlein et al., 1984; Facer et al., 1993; Avdeev et al., 2002). The iridium pyrochlore Pb_2_Ir_2_O_6.5_ (Kennedy, 1996) exhibits similar vacancy ordering and its structure is also described in $F\bar{4}3m$ however this vacancy ordered structure was not seen in Pb_2_Re_2_O_7-d_ (Abakumov et al., 1998). It is generally believed that Re is a suitable, non-radioactive, analogue for Tc.

Given the prevalence of oxygen non-stoichiometry in pyrochlores it is probable that, as suggested by Muller et al., Pb_2_Tc_2_O_7-d_ will be non-stoichiometric, however it is unclear if oxygen vacancy ordering will occur. The aim of the present work was to determine the structure of the lead-technetium pyrochlore, and in particular to establish the nature of any non-stoichiometry. This has been accomplished using a combination of synchrotron X-ray and neutron powder diffraction methods. As we show here the target oxide is indeed non-stoichiometric, however we find no evidence for oxygen-vacancy ordering. X-ray absorption near-edge structure (XANES) at the Tc K-edge and Raman spectroscopy measurements are also reported.

## Experimental

*Caution!* ^99^ *Tc is a β* ^*-*^ *emitter (E*_*max*_ *= 0.29 MeV). Appropriate shielding was employed during the synthesis and all manipulations.* The polycrystalline sample of Pb_2_Tc_2_O_7-d_ was prepared by the addition of 2.55 g of NH_4_TcO_4_ to 2.34 g of Pb(NO_3_)_2_, this mixture was then dry rolled in a polyethylene vial for 2 h to ensure complete mixing, the powder was then calcined in Ar for 1 h at 650°C. The Ar used had 12 ppm of O_2_, this percentage of O_2_ needs to be taken into account when calcining or sintering these samples as extended exposure to even small amounts of O_2_ will cause the Tc to oxidise and sample inhomogeneity will occur. The resultant calcine melted and so was ground in a mortar and pestle, wet ball milled in cyclohexane for 16 h and then tray dried. Note that milling in any other types of fluids may result in loss of Tc. Two one-gram pellets were pressed and sintered in Ar for 4 h at 800°C.

The synchrotron X-ray diffraction data were collected using the MYTHEN microstrip detector on the powder diffractometer at BL-10 of the Australian Synchrotron, Melbourne Australia (Wallwork et al., 2007). The sample (ca 1 mg) was loaded into a 0.3 mm diameter glass capillary inside a glovebox. The sealed capillary was rotated during the collection of the X-ray diffraction data. The data were recorded at room temperature in the angular range 5 < 2θ < 85°, using X-rays of wavelength 0.82523 Å as estimated using NIST LaB_6_. For high temperature measurements the sample was loaded into a 0.3 mm quartz capillary inside a glovebox and heated via a Cyberstar hot-air blower to up to 1,000°C. Neutron powder diffraction data were measured at room temperature using the high-resolution powder diffractometer Echidna at ANSTO’s OPAL facility at Lucas Heights (Avdeev and Hester, 2018). These measurements were taken at *λ* = 1.540 Å, with the sample ( ∼1 g) contained in a cylindrical vanadium can. The structure refinement used a combination of the synchrotron and neutron diffraction data sets using the program RIETICA (Liss et al., 2006).

X-ray absorption near-edge structure (XANES) spectra were collected from the sample as well as the (NH_4_)TcO_4_ and SrTcO_3_ standards at the Tc K-edge on beamline BL-12 (Glover et al., 2007) at the Australian Synchrotron in transmission mode using argon-filled ionization chambers (Blanchard et al., 2014). A total of 2 mg of each Tc-containing powder sample was first mixed with an appropriate amount of BN, and the mixture was then loaded into a 3.5-mm-diameter hole at the center of a 1-mm-thick aluminum plate. The samples were sealed using Kapton tapes on both sides of the aluminum plate. The energy calibration was carried out using the Mo K-edge at 20,000 eV, steps of 0.2 eV were used across the edge. The software package Athena was used for background subtraction and normalization (Ravel and Newville, 2005).

Electron diffraction patterns and microanalyses were obtained at ANSTO using a JEOL 2000FXII TEM operated at 200 kV and equipped with a Link ISIS ultra-thin window solid-state Si(Li) detector and microanalysis system. The TEM was calibrated for electron diffraction work over a range of objective lens settings using a polycrystalline gold standard. The sample was ground and loaded onto a copper grid for analysis.

Raman spectra were obtained using a Renishaw inVia Qontor confocal Raman microscope (Renishaw plc., Wotton-under-Edge, United Kingdom) with a 532 nm continuous wave, diode-pumped solid-state laser (Renishaw plc., Wotton-under-Edge, United Kingdom). The measurements were carried out with 0.1 mW of laser power on the sample and a x50/0.5NA long working distance objective, giving rise to a focused spot of approximately 1.3 µm diameter. The scattered light was analysed in backscattering geometry using holographic notch filters, 2,400 lines/mm grating and an air-cooled CCD detector. The spectra were collected at temperatures 298 K (25°C), 273 K (0°C) and 100 K (−173°C) using a FTIR600 variable-temperature stage (Linkam Scientific Instruments Ltd, Surrey, United Kingdom). Data collection was performed over a spectral range of 89–1,348 cm^-1^ with 100 accumulations/data set of 5 s exposure time per accumulation and an equilibration time of 10 min at each temperature. To avoid potential contamination of the equipment while maximizing temperature conductivity from the variable-temperature plate to the sample, the powder sample (ca 1 mg) was loaded into a 0.3 mm diameter quartz capillary inside a glovebox and this was sealed between two copper plates using thermal paste. An aperture in the top plate enabled the laser to reach the sample.

## Results and Discussion

### Diffraction Studies

The structure of Pb_2_Tc_2_O_7-*d*_ was initially refined as a cubic pyrochlore in space group $Fd\bar{3}m$ (Number 227) (Subramanian et al., 1983) with the Pb on the 16*d* sites, Tc on the 16*c*; O(1) on 48*f* and O(2) on 8*b*. In this structure there is only one variable fractional coordinate, for O(1) at (*x*, 1/8, 1/8) with *x* ∼ 0.3. The unit cell parameter 10.36584(2) Å was determined in the Rietveld refinement using the combination of synchrotron X-ray and neutron diffraction data. This value is in excellent agreement with that reported by Muller et al. (1964) (10.361 Å). Examination of the diffraction profiles revealed weak reflections due to the presence of small amounts of an unidentified impurity phase when the *y* axis was plotted as a sqrt of counts, and they are not visible in the difference plot of counts vs. angle. These phases could not be identified because there was not a sufficient number of peaks to match with known starting elements. Upon heating these reflections disappeared and so they are assumed to be a small amount of unreacted starting material therefore such reflections were excluded from the Rietveld refinements. Refinement of the pattern collected at 1,000°C resulted in a unit cell parameter of 10.45961(5) Å with no phase change visible, the increase in unit cell parameter consistent with thermal expansion. The refinements, using X-ray or neutron diffraction data or a combination of both, gave larger than expected atomic displacement parameters [B_iso_ = 3.4(1) Å^2^ for the O(2) atoms at (3/8 3/8 3/8)] which is indicative of vacancies. Refinement of the occupancy for this site yielded a value 0.86(4) with the displacement parameter reducing somewhat to 2.6(1) Å^2^. There was no evidence for reflections of the type *hk*0 with *h* + *k* = 2*n* such as the 420 or 640 reflections in the diffraction patterns indicative of a lowering of symmetry from $Fd\bar{3}m\;$ to $F\bar{4}3m$ as is observed for Pb_2_Ru_2_O_6.5_ (Beyerlein et al., 1984) and Pb_2_Ir_2_O_6.5_ (Kennedy, 1996). Evidently the structure of Pb_2_Tc_2_O_7-d_, like that of Pb_2_Re_2_O_7-d_ (Abakumov et al., 1998), is described by space group $Fd\bar{3}m$ and does not exhibit oxygen vacancy ordering.

Previous high resolution structural studies of pyrochlores containing Bi cations on the *A*-site including Bi_2_Tc_2_O_7-d_ (Rodriguez et al., 2008), have revealed static disorder of the Bi cations (Vanderah et al., 2005; Somphon et al., 2006). It should be noted that this is in contrast to the study of Ln_2_Tc_2_O_7_ compounds performed by Hartman and co-workers (Hartmann et al., 2011), however such disorder has also been seen in La_2_Zr_2_O_7_ (Tabira et al., 2001); although it is amplified by the presence of the Bi 6s^2^ lone pair electrons (Vanderah et al., 2005). Given that Pb^2+^ is isoelectronic with Bi^3+^, the possibility of static disorder of the Pb cations was then considered. Two models, where the Pb was displaced from the 16*d* site to either a 96*g* or 96*h* site, were explored. Both models resulted in a small improvement in the quality of the fit, however the data did not allow us to distinguish the best model between these. These two models are effectively equivalent, and we present here the results obtained with the Pb disordered on the 96*h* site. The possibility that the O(2) atom was also disordered on the 32*e* site was considered and the inclusion of such disorder resulted in a noticeable reduction in the displacement parameter for this atom to 0.71(23) Å^2^. There is a noticeable difference in the values of the B_iso_ between the Pb and the Tc, this is due to the Biso for the 8-coordinate site in the pyrochlores being larger than that of the 6-coordinate site reflecting its more irregular environment. Interestingly the electron diffraction images of Pb_2_Re_2_O_7-d_ published by Abakumov and co-workers (Abakumov et al., 1998) did not show any diffuse features characteristic of cation disorder suggesting there may be subtle differences between the Re and Tc oxides. Diffuse features have been observed in electron diffraction studies of numerous Bi containing pyrochlores including Bi_2_Ru_2_O_7_ (Goodwin et al., 2007).

**FIGURE 1:**
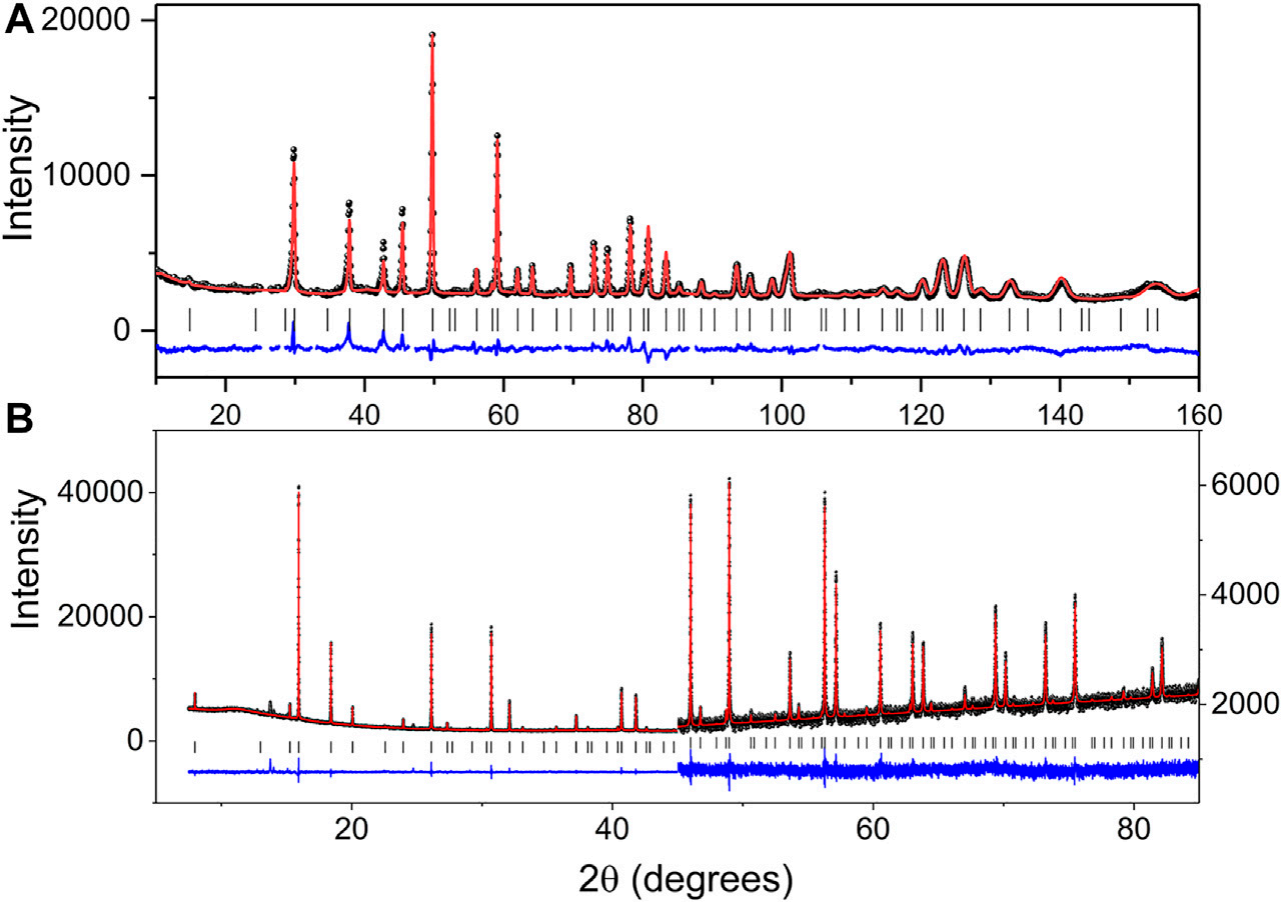
Observed, calculated and difference **(A)** neutron and **(B)** synchrotron X-ray diffraction profiles (second half of the pattern has a scale increase of 8.3 to allow the difference and observed profiles to align) for Pb_2_Tc_2_O_7-d_. The change in scale near 2θ = 45° in the SXRD pattern highlights both the quality of the data and fit.

The results of the refinements are summarised in Table 1 and are illustrated in Figure 1.

**TABLE 1:** Refined atomic coordinates and atomic displacement parameters for Pb_2_Tc_2_O_7-d_. These parameters were obtained by refinement against a combined neutron and synchrotron X-ray diffraction data set. *a* = 10.36581(2)Å R_P_ = 2.50 R_WP_ = 3.46% χ^2^ = 12.53. A comparably good fit could be obtained if the Pb was placed on the 96*h* site.

Atom	Site	*x*	*y*	*z*	B_iso_	N
Pb	96*h*	0	0.2523(14)	0.7477(14)	0.60(4)	2^a^
Tc	16*c*	0	0	0	0.07(2)	2
O(1)	48*f*	0.3189(1)	0.125	0.125	0.30(1)	6
O(2)	32*e*	0.3918(5)	0.3918(5)	0.3918(5)	0.71(23)	0.86(1)

a N is the equivalent number of atoms present, the Pb is on the 96*h* site and shows six-fold disorder.

**FIGURE 2:**
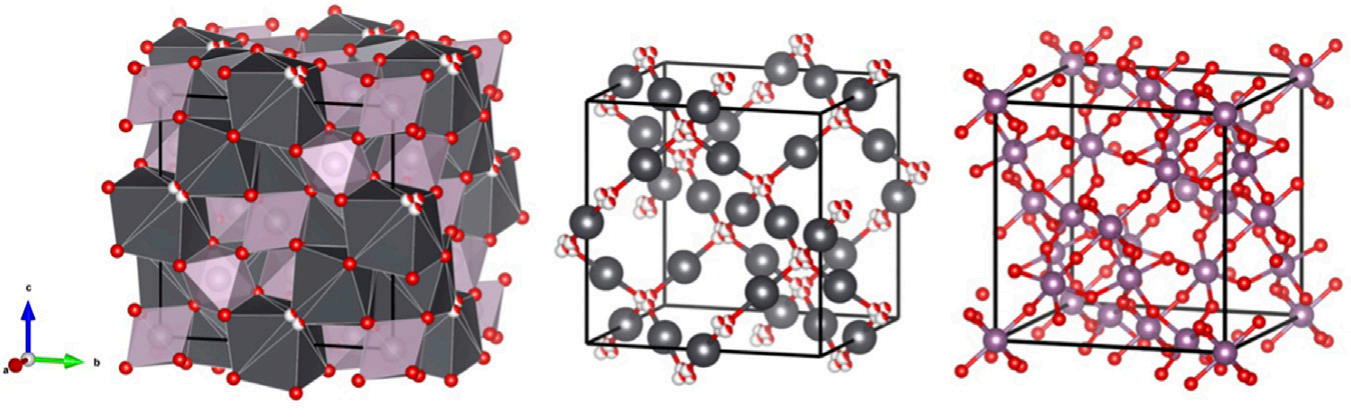
Interconnecting network and the two separate sub lattices, Pb sites are shown in grey, Tc sites in purple, O(1) as fully coloured (red) spheres, and O(2) are red and white spheres, indicating the four-fold disorder of these.

The wide-spread prevalence of oxygen vacancies in pyrochlore oxides is rationalised by viewing the structure as based on two weakly interacting, but interpenetrating networks of formula Pb_2_O(2) and Tc_2_O(1)_6_ as illustrated in Figure 2.

The Pb cations in Pb_2_Tc_2_O_7-d_ are in a compressed scalenohedral environment. The displacement of the Pb cations within the puckered hexagon of the PbO(1) group reduces two Pb-O(1) distances but increases the remaining four. The average Pb-O distance of 2.625(8) Å is not significantly changed from that seen in the ideal structure and is much longer than the two Pb-O(2) bonds along the $\bar{3}$ axis, 2.244(1) Å, reflecting a relatively weak interaction between the Pb and the Tc_2_O_6_ network. Presumably the displacement of the Pb is a response to the abnormally high bond valence that would occur if the Pb remained on the 16*d* site, viz 2.79. The BVS estimated for the Pb assuming the O(2) remains at the 8*a* site is 1.95. The Tc cations are surrounded by six O(1) atoms in a trigonal antiprism geometry with six equal Tc-O(1) distances of 1.9672(4) Å. This distance is shorter than the average Tc-O distances of 1.983 and 1.997 Å seen in SrTcO_3_ and CaTcO_3_ and of 2.011 Å reported for Bi_2_Tc_2_O_7-d,_ where the Tc is formally tetravalent. The BVS for Tc is estimated to be 4.48 using the R_o_ value of 1.859 Å reported for Tc^5+^ by Wester and Hess (2005). The Tc-O(1)-Tc angle is 137.34(6)°. This angle is appreciably larger than the 134.109(4)° found for Bi_2_Tc_2_O_7-d_ (Rodriguez et al., 2008). In general this bond angle is larger in the Pb pyrochlores than those seen in the corresponding Bi pyrochlores; 134.81 vs. 133.17° for Pb_2_Ru_2_O_6.5_ (Beyerlein et al., 1984) and Bi_2_Ru_2_O_7_ (Facer et al., 1993; Avdeev et al., 2002) respectively and 133.27 vs. 131.39° for the analogous Ir oxides (Kennedy, 1996). Given that these Ru and Ir pyrochlores are metallic oxides, we anticipate that Pb_2_Tc_2_O_7-d_ will also be metallic. The magnitude of the Tc-O(1)-Tc bond angle has been associated with the presence of metallic bonding in Ru pyrochlores. The incorporation of Pb^2+^ or Bi^3+^ on the *A*-site increases this bond angle and promotes metallic bonding, however semi-conducting behavior has been observed when lanthanoid cations occupy the *A*-site (Kennedy and Vogt, 1996). Reported DFT calculations of the rare earth technetate pyrochlores suggest that these will be metallic, and although the Tc-O(1)-Tc angle in such oxides is unknown we speculate that it will be greater than that seen in the corresponding, semiconducting ruthernates.

**FIGURE 3:**
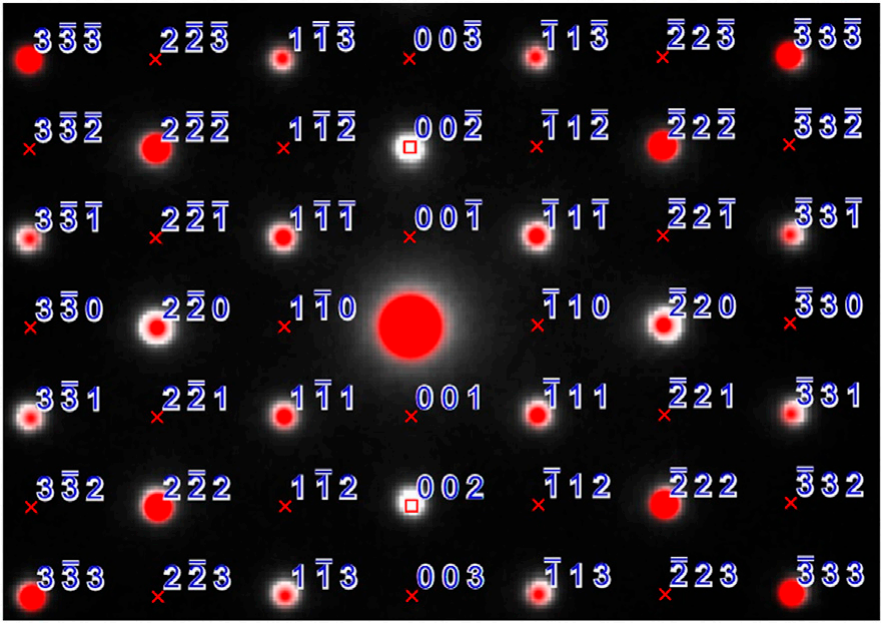
SAD pattern with experimental data shown as white dots, red dots indicate the simulated pattern, reflections are marked, lattice absences are shown with a red cross and space group absences are shown with a red square.

Selected area diffraction patterns (SAD) were indexed with the SingleCrystal™ software (CrystalMaker, 2021) *via* the coordinates obtained from X-ray and neutron diffraction analysis. The experimental patterns were in good agreement with the predicted patterns as shown in Figure 3, the zone axis for the experimental and simulated SAD patterns is [110]. An overlay of the simulated SAD patterns on the experimental one shows crosses for the forbidden reflections that are not shown in experimental image except {002} due to double diffraction. For the space group $Fd\bar{3}m$ {002} reflections when viewed down the [110] axis are kinematically forbidden. If the TEM specimen is sufficiently thick those reflections arise due to double scattering (double diffraction) by the ±(−111) and ±(1–11) reflections (Liao, 2006).

### X-ray Absorption Spectroscopy

**FIGURE 4:**
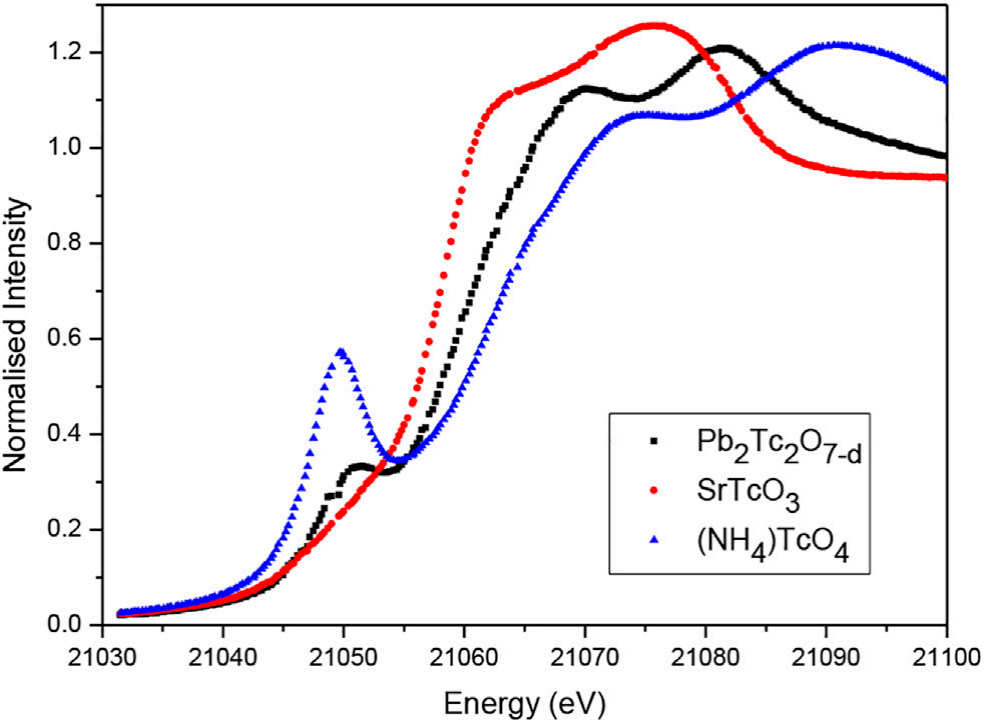
Normalised X-ray absorption spectra of Pb_2_Tc_2_O_7-d_, SrTc^4+^O_4_ and (NH_4_)Tc^7+^O_4_. The Tc has six-fold coordination in Pb_2_Tc_2_O_7-d_ and SrTcO_3_ and is in a tetrahedral environment in (NH_4_)TcO_4_.

The X-ray absorption spectrum of Pb_2_Tc_2_O_7-d_ in the region of the Tc K-edge is dominated by a pronounced 1s - 5p dipole allowed transition at about 21,058 eV. This is illustrated in Figure 4, together with the spectra of SrTcO_3_ and (NH_4_)TcO_4_. It is immediately apparent from this figure that the energy of the Tc K-edge in Pb_2_Tc_2_O_7-d_ is intermediate between that of the Tc^4+^ (SrTcO_3_) and Tc^7+^ ((NH_4_)TcO_4_) standards. This is consistent with formal valence of Tc being greater than four as indicated by the BVS calculations. Using the stoichiometry derived from the neutron diffraction measurement, Pb_2_Tc_2_O_7-d_, and assuming the lead remains divalent, Tc has a formal charge of +4.86.

The presence of the strong pre-edge feature in the spectrum of Pb_2_Tc_2_O_7-d_ is somewhat unexpected. Such a feature is not apparent in the published spectra of Bi_2_Tc_2_O_7-d_ (Rodriguez et al., 2008). The intensity of the pre-edge feature, seen in many K-edge spectra, is known to be sensitive to the site symmetry of the absorber. The transition may be assigned to a, formally dipole forbidden in centrosymmetric species, 1s → 4d transition. This transition is weakly quadrupole-allowed and gains intensity by mixing of the metal p-orbitals in a non-centrosymmetric absorber (Laplaza et al., 1996). This is clearly evident in the spectrum of tetrahedral (NH_4_)TcO_4_ where the 1s → 4d transition at 21047.7 eV is observed to be relatively intense. Studies of various Mo and Ru compounds demonstrate that well resolved pre-edge features can be observed for six-coordinate complexes where the symmetry is not strictly octahedral (Laplaza et al., 1996; Resseler et al., 2000; Sikora et al., 2007) and it is possible that this is the case here. An alternate possibility, that the sample has partially decomposed to produce a lower-symmetry material during the measurements, cannot be discounted. Irrespective of the origin of this feature in the Tc K-edge the XAS demonstrates the Tc oxidation state to be greater than 4+.

### Raman Spectroscopy

Raman spectra of the Pb_2_Tc_2_O_7-δ_ sample were collected at 298 K (25°C), 273 K (0°C) and 100 K (−173°C). To correct for the Bose-Einstein occupation factor (Loudon, 1964), the raw data was divided by *n*(ω)+1, where *n*(ω) is the Bose-Einstein distribution given by:

$$n\left(\omega \right)=\frac{1}{e^{\hbar \omega /k_{B}T}-1}$$ (1)

Here, ℏ is the reduced Plank’s constant, $k_{B}$ is the Boltzmann constant, T is the absolute temperature and ω is the angular frequency, which is related to the Raman shift according to Eq. 2:

ω=2πc(Raman shift) (2)

where c is the speed of light.

**FIGURE 5:**
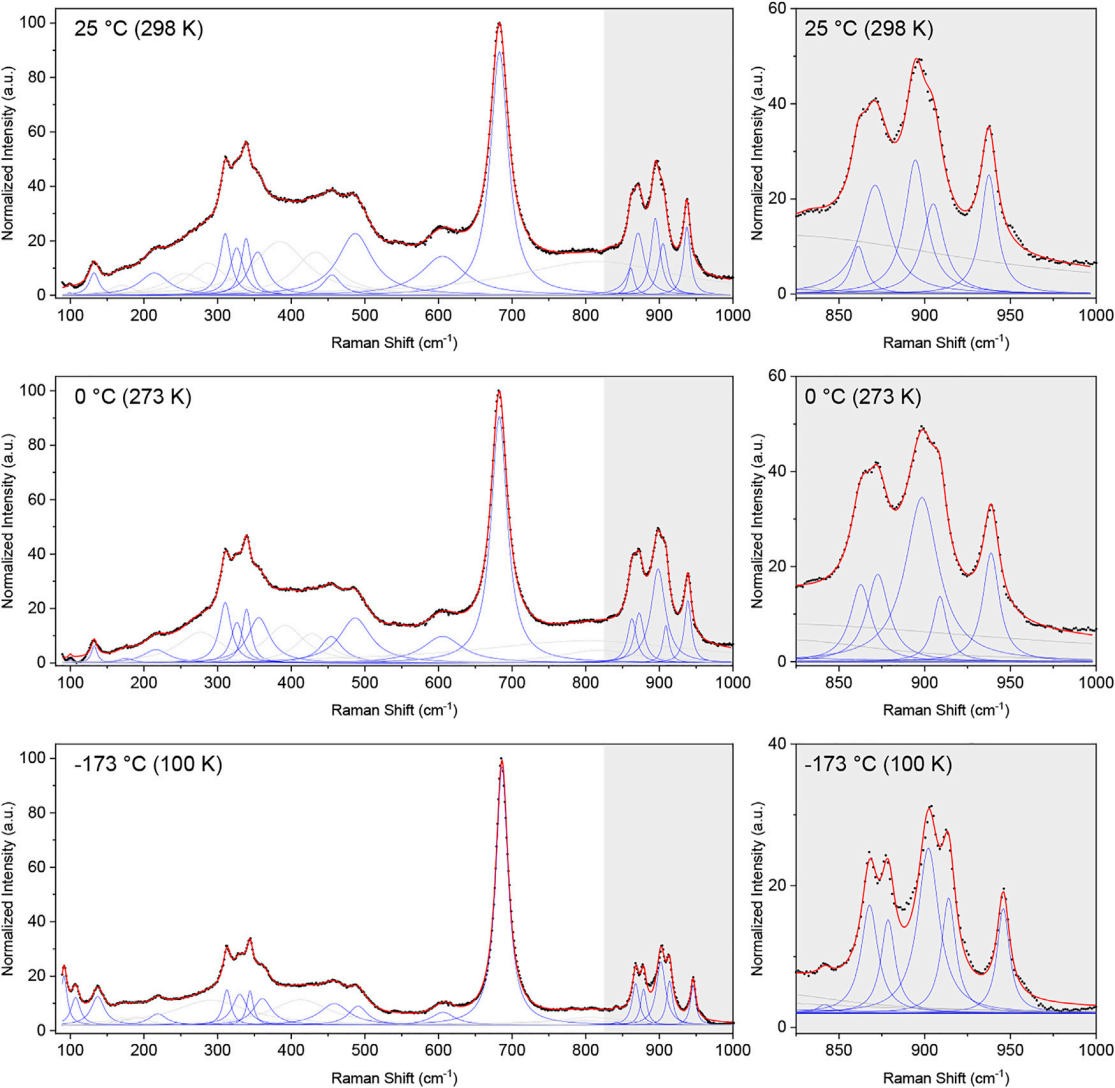
Normalized Raman spectra corrected for the Bose-Einstein population factor (black dots) and cumulative fit (red line) of a sum of Lorentzian functions (blue and grey) to the Raman spectra collected at 298 K (25°C), 273 K (0°C) and 100 K (−173°C). The broader Lorentzian peaks, which do not describe a defined peak but contribute to the description of the background are shown in grey, whereas peaks describing clearly visible peaks are shown in blue. An enhancement of the region between 825 and 1,000 cm^-1^ is given for each temperature, emphasizing the narrower nature of the peaks at lower temperatures.

The corrected intensities were normalized by the maximum value and fitted over the range of 100–1,000 cm^-1^ using a sum of Lorentzian functions. The obtained spectra at 298, 273, and 100 K are shown in Figure 5. The spectra comprise a large number of overlapping and convoluted Raman modes, and several Lorentzian peaks (>23 peaks) were needed in order to obtain a satisfactory fit of the data (see Supplementary Material for details of the fits). The most significant fitted bands (*i.e.,* those describing clear, “sharp” peaks in the data) are shown in blue in Figure 5, whereas broader peaks describing background features are shown in light grey. The same general features are observed in the spectra measured at each of the three temperatures, although the observed sharpening of the peaks at lower temperatures emphasises the doublet nature of some peaks (see grey insert of Figure 5).

The ideal pyrochlore structure in space group $Fd\bar{3}m$, with no cationic or anionic disorder, gives rise to six Raman-active modes, seven IR modes and one acoustic mode, according to factor group analysis (McCauley, 1973; Glerup et al., 2001). The six Raman-active modes given per set of ions and their respective crystallographic positions are:

A (16d): none

B (16c): none

$$\text{O}\left(1\right)\;\left(48f\right):\;A_{g}+E_{g}+3F_{2g}$$

$$\text{O}\left(2\right)\;\left(8b\right):\;F_{2}g$$

The data unequivocally shows the presence of more than the six Raman bands expected for the ideal pyrochlore structure. In disordered structures, additional bands may emerge due to a change in local symmetry or a breakdown of the selection rules leading to silent and IR-active modes to appear in the Raman spectrum (Glerup et al., 2001). In the case of the disordered Pb_2_Tc_2_O_7-d_ studied here, with the Pb and O(2) anions occupying the 96*h* and 32*e* Wyckoff positions, respectively, group theory predicts a total of 17 Raman-active modes, given by (Kroumova et al., 2003):

$$\text{Pb}\;\left(96h\right):A_{1g}+3E_{g}+4F_{2g}$$

Tc (16c): none

$$\text{O}\left(1\right)\;\left(48f\right):\;A_{1g}+E_{g}+3F_{2g}$$

$$\text{O}\left(2\right)\;\left(32e\right):\;A_{1g}+E_{g}+2F_{2g}$$

The number of predicted Raman-active modes agrees well with the number of significant peaks needed to obtain a satisfactory fit to the data (*i.e.,* 17 significant fitted peaks in the 100 K Raman spectrum and 16 in the other two spectra), however, given the complex nature of the spectra we cannot confidently assign the fitted peaks to correspond to the aforementioned 17 Raman-active bands predicted for a disordered pyrochlore. Nevertheless, the data clearly shows the presence of several additional modes than that of the archetypical pyrochlore structure, corroborating the displacive disorder of the Pb and O(2) sites in Pb_2_Tc_2_O_7-d_. A similar observation was made by Arenas et al. (2010) in their Raman study of substituted bismuth pyrochlores, where displacive disorder gave rise to additional Raman bands.

## Conclusion

The structure of lead-technetium pyrochlore has been refined using a combined synchrotron X-ray and neutron powder diffraction data set. The oxide is found to be oxygen deficient with a stoichiometry of Pb_2_Tc_2_O_7-d_ with disordered oxygen vacancies. In this regards the structure is similar to Pb_2_Re_2_O_7-d_ rather than to the analogous ruthenate, which displays ordering of the oxygen vacancies. Displacive disorder of the Pb cations and O(2) anions is observed, as evident from the refinements. X-ray absorption spectroscopic measurements at the Tc K-edge demonstrate the valence of the Tc is greater than 4+. Taking into account the BVS results from diffraction analysis, the most likely conclusion is that Tc is in a 5+ oxidation sate. Raman spectroscopy confirmed a change in the local structure and coordination of the ions compared to the ideal, non-disordered pyrochlore structure, supporting the displacive disorder present in Pb_2_Tc_2_O_7-d_.

## Data Availability

The original contributions presented in the study are included in the article/Supplementary Material, further inquiries can be directed to the corresponding author.
