# Supplementary material for: Synthesis and Structure of Oxygen Deficient Lead-Technetium Pyrochlore, the First Example of a Valence V Technetium Oxide
Source: Front Chem. 2021 Jul 1;9:706269. doi: 10.3389/fchem.2021.706269 (PMC8281135; doi:10.3389/fchem.2021.706269)
Supplement: Supplementary file 1 [file DataSheet1.docx]

# Additional figures and tables for Supporting Information

Plots of the Lorentzian fits to the Raman spectra and tables containing the numerical values from the fits are given below for the spectra collected at the three temperatures (room temperature, 0 °C and -173 °C).

## Multipeak fit of imaginary part of Raman susceptibility by a sum of Lorentzian functions

The fitting of the data was performed with the software OriginPro using the following Lorentzian equation:

$$y=y_{0}+\frac{2A}{\pi} \frac{w}{{4\left( x-x_{c} \right)}^{2}+w^{2}}$$

Where $y_{0}$ is the offset, $x_{c}$ is the centre frequency, $w$ is the peak width and $A$ is the area of the peak. The height of the peak is given by:

$$H=\frac{2A}{\pi w}$$

The numeric values extracted from the fitting of the three datasets are given below.

## RT spectrum – Lorentzian fit parameters

|  | **y_0_** | | **x_c_** | | **w** | | **A** | | **H** | |
| --- | --- | --- | --- | --- | --- | --- | --- | --- | --- | --- |
|  | **Value** | **Standard Error** | **Value** | **Standard Error** | **Value** | **Standard Error** | **Value** | **Standard Error** | **Value** | **Standard Error** |
| **Peak1** | 0 | 0 | 99 | 0 | 5 | 0 | 8.53745 | 3.40443 | 1.08702 | 0.43347 |
| **Peak2** | 0 | 0 | 132.2445 | 0.30916 | 15.77596 | 1.22572 | 205.0342 | 17.81852 | 8.27391 | 0.32875 |
| **Peak3** | 0 | 0 | 169.7609 | 1.81162 | 40.74091 | 8.43801 | 234.092 | 66.5102 | 3.65794 | 0.41744 |
| **Peak4** | 0 | 0 | 213.8963 | 2.05176 | 47.36332 | 9.83769 | 611.4312 | 290.2071 | 8.21837 | 2.30453 |
| **Peak5** | 0 | 0 | 255.2473 | 13.97577 | 64.28329 | 49.80521 | 801.4044 | 1816.857 | 7.93659 | 12.05658 |
| **Peak6** | 0 | 0 | 286.4327 | 7.98904 | 62.75764 | 48.03993 | 1168.366 | 2023.418 | 11.85202 | 11.63232 |
| **Peak7** | 0 | 0 | 310.416 | 0.35227 | 18.18643 | 2.33899 | 646.3394 | 197.062 | 22.62525 | 4.08971 |
| **Peak8** | 0 | 0 | 326.0225 | 0.82768 | 21.95919 | 5.22401 | 601.2538 | 259.251 | 17.43097 | 3.69861 |
| **Peak9** | 0 | 0 | 338.7676 | 0.41258 | 16.10578 | 2.59035 | 528.1041 | 190.8683 | 20.87458 | 4.32269 |
| **Peak10** | 0 | 0 | 354.3741 | 1.11012 | 30.44802 | 7.25353 | 761.1473 | 456.6856 | 15.91438 | 5.90694 |
| **Peak11** | 0 | 0 | 384.7031 | 5.60841 | 87.71221 | 44.08608 | 2717.252 | 2735.947 | 19.72195 | 10.10313 |
| **Peak12** | 0 | 0 | 432.784 | 5.90399 | 69.67172 | 47.00616 | 1733.919 | 2616.7 | 15.84355 | 13.30985 |
| **Peak13** | 0 | 0 | 455.3606 | 1.42933 | 29.21602 | 12.89512 | 344.6958 | 465.3636 | 7.51095 | 6.91568 |
| **Peak14** | 0 | 0 | 486.9041 | 1.04128 | 57.22984 | 3.47367 | 2039.507 | 401.2391 | 22.6873 | 3.32166 |
| **Peak15** | 0 | 0 | 546.3211 | 1.21206 | 41.47332 | 6.3052 | 283.8297 | 63.47938 | 4.35682 | 0.42484 |
| **Peak16** | 0 | 0 | 605.5425 | 0.54377 | 71.30257 | 2.27085 | 1605.777 | 63.28337 | 14.33706 | 0.2453 |
| **Peak17** | 0 | 0 | 682.7354 | 0.03457 | 29.5046 | 0.14124 | 4143.2 | 20.8833 | 89.39768 | 0.21488 |
| **Peak18** | 0 | 0 | 813.8256 | 1.97655 | 275.4133 | 5.82429 | 5371.368 | 109.8723 | 12.41596 | 0.14712 |
| **Peak19** | 0 | 0 | 833.3359 | 1.72318 | 18.1738 | 0 | 40 | 0 | 1.40118 | 0 |
| **Peak20** | 0 | 0 | 861.2899 | 0.3229 | 10.12369 | 1.43242 | 159.7968 | 42.5772 | 10.04869 | 1.39802 |
| **Peak21** | 0 | 0 | 871.0764 | 0.47112 | 20.41057 | 1.05885 | 734.4072 | 65.58813 | 22.90667 | 0.99855 |
| **Peak22** | 0 | 0 | 894.6445 | 0.10751 | 15 | 0 | 665.0691 | 8.49108 | 28.22641 | 0.36037 |
| **Peak23** | 0 | 0 | 905 | 0 | 15 | 0 | 447.3719 | 8.61516 | 18.98705 | 0.36564 |
| **Peak24** | 0 | 0 | 937.4592 | 0.06977 | 11.42069 | 0.2453 | 450.3484 | 8.51943 | 25.10361 | 0.31024 |

## 0 °C (273 K) spectrum – Lorentzian fit parameters

|  | **y_0_** | | **x_c_** | | **w** | | **A** | | **H** | |
| --- | --- | --- | --- | --- | --- | --- | --- | --- | --- | --- |
|  | **Value** | **Standard Error** | **Value** | **Standard Error** | **Value** | **Standard Error** | **Value** | **Standard Error** | **Value** | **Standard Error** |
| **Peak1** | 0 | 0 | 100 | 0 | 5 | 0 | 10 | 0 | 1.27324 | 0 |
| **Peak2** | 0 | 0 | 132.4551 | 0.25769 | 9.56191 | 0.81923 | 93.75237 | 6.42077 | 6.24191 | 0.33593 |
| **Peak3** | 0 | 0 | 175 | 0 | 26.52577 | 6.98013 | 69.56789 | 19.17367 | 1.66963 | 0.23562 |
| **Peak4** | 0 | 0 | 216.1446 | 1.07311 | 42.38747 | 5.26161 | 326.4531 | 57.21607 | 4.90302 | 0.36513 |
| **Peak5** | 0 | 0 | 276.8179 | 1.42706 | 74.69283 | 6.84395 | 1323.596 | 163.8642 | 11.28124 | 0.49506 |
| **Peak6** | 0 | 0 | 310.4525 | 0.33262 | 19.49638 | 1.31028 | 679.8411 | 89.55525 | 22.199 | 1.55664 |
| **Peak7** | 0 | 0 | 326.1567 | 0.54546 | 19.83779 | 3.52226 | 466.0077 | 131.4334 | 14.95478 | 1.65392 |
| **Peak8** | 0 | 0 | 339.3048 | 0.28003 | 14.6183 | 1.81365 | 453.7939 | 117.4303 | 19.7625 | 2.76714 |
| **Peak9** | 0 | 0 | 355.9327 | 1.00155 | 36.32158 | 7.62706 | 949.191 | 414.7631 | 16.63677 | 3.87526 |
| **Peak10** | 0 | 0 | 391.4498 | 4.25803 | 66.56088 | 28.48416 | 1439.126 | 1295.585 | 13.76449 | 6.6191 |
| **Peak11** | 0 | 0 | 428.0832 | 3.66777 | 55.3395 | 32.85915 | 935.2432 | 1268.059 | 10.75894 | 8.28032 |
| **Peak12** | 0 | 0 | 454.2738 | 2.01946 | 36.88115 | 10.84386 | 561.7921 | 450.6837 | 9.69731 | 5.04714 |
| **Peak13** | 0 | 0 | 486.8553 | 1.03924 | 51.97076 | 3.3354 | 1348.663 | 147.0706 | 16.52055 | 0.85397 |
| **Peak14** | 0 | 0 | 544.7285 | 1.8138 | 48.78253 | 9.33028 | 255.4128 | 69.02078 | 3.33318 | 0.35604 |
| **Peak15** | 0 | 0 | 605.5152 | 0.70413 | 64.85147 | 2.78684 | 996.5851 | 53.88751 | 9.78306 | 0.22288 |
| **Peak16** | 0 | 0 | 682.5084 | 0.03035 | 26.69854 | 0.13275 | 3796.56 | 21.1746 | 90.52798 | 0.2283 |
| **Peak17** | 0 | 0 | 800 | 0 | 371.8759 | 31.42784 | 4743.346 | 579.221 | 8.1202 | 1.5352 |
| **Peak18** | 0 | 0 | 818.5278 | 4.07255 | 132.521 | 36.75737 | 969.0721 | 571.0317 | 4.65534 | 1.48832 |
| **Peak19** | 0 | 0 | 862.7176 | 0.42033 | 14.79326 | 1.15503 | 377.3822 | 61.04316 | 16.24043 | 1.52302 |
| **Peak20** | 0 | 0 | 872.5983 | 0.41214 | 15.69804 | 1.2112 | 453.3968 | 64.71266 | 18.38709 | 1.36352 |
| **Peak21** | 0 | 0 | 898.393 | 0.31802 | 23.29666 | 0.8701 | 1264.594 | 78.68706 | 34.55711 | 0.9688 |
| **Peak22** | 0 | 0 | 908.8859 | 0.18736 | 9.98161 | 1.06947 | 215.1099 | 42.15088 | 13.71956 | 1.3329 |
| **Peak23** | 0 | 0 | 938.7156 | 0.07557 | 11.61856 | 0.27656 | 417.4618 | 9.43128 | 22.87414 | 0.29678 |

## -173 °C (100 K) spectrum – Lorentzian fit parameters

|  | **y_0_** | | **x_c_** | | **w** | | **A** | | **H** | |
| --- | --- | --- | --- | --- | --- | --- | --- | --- | --- | --- |
|  | **Value** | **Standard Error** | **Value** | **Standard Error** | **Value** | **Standard Error** | **Value** | **Standard Error** | **Value** | **Standard Error** |
| **Peak1** | 2 | 0 | 91.33601 | 0.18463 | 10.50478 | 0.90943 | 298.0869 | 26.05408 | 18.06492 | 0.51336 |
| **Peak2** | 2 | 0 | 107.16 | 0.35635 | 14.91172 | 1.44395 | 238.5739 | 27.87505 | 10.18534 | 0.5123 |
| **Peak3** | 2 | 0 | 137.2158 | 0.35971 | 21.87395 | 1.79353 | 360.9624 | 42.87032 | 10.50546 | 0.6306 |
| **Peak4** | 2 | 0 | 169.8772 | 4.28218 | 24.04972 | 20.15147 | 100.6241 | 212.6101 | 2.66362 | 3.5141 |
| **Peak5** | 2 | 0 | 189.3371 | 10.46382 | 40.32642 | 50.88706 | 188.7144 | 401.3237 | 2.97917 | 2.67559 |
| **Peak6** | 2 | 0 | 218.762 | 2.20221 | 28.51079 | 12.6377 | 188.4443 | 165.9949 | 4.20779 | 1.95199 |
| **Peak7** | 2 | 0 | 244.8762 | 6.60578 | 23.40648 | 12.87227 | 30 | 0 | 0.81595 | 0.44873 |
| **Peak8** | 2 | 0 | 291.4881 | 11.93924 | 123.3094 | 44.54983 | 1782.389 | 873.292 | 9.20209 | 1.30955 |
| **Peak9** | 2 | 0 | 312.5377 | 0.29601 | 13.06453 | 1.61879 | 266.3586 | 56.41477 | 12.97936 | 1.29963 |
| **Peak10** | 2 | 0 | 330.0788 | 0.82701 | 24.25326 | 5.45573 | 431.8339 | 152.0046 | 11.33513 | 1.91242 |
| **Peak11** | 2 | 0 | 343.9549 | 0.28778 | 10.73954 | 1.63306 | 213.617 | 56.14737 | 12.66281 | 1.5919 |
| **Peak12** | 2 | 0 | 360.7011 | 0.96242 | 30.44582 | 5.23478 | 468.7135 | 148.036 | 9.80077 | 1.62102 |
| **Peak13** | 2 | 0 | 412.643 | 5.37486 | 97.09981 | 29.32665 | 1430.179 | 726.6191 | 9.37675 | 2.12158 |
| **Peak14** | 2 | 0 | 458.5807 | 1.81653 | 48.93201 | 12.98867 | 611.2555 | 334.4911 | 7.95261 | 2.35401 |
| **Peak15** | 2 | 0 | 490.7427 | 1.16245 | 31.64234 | 4.18641 | 348.4433 | 80.69332 | 7.01041 | 0.83431 |
| **Peak16** | 2 | 0 | 549.61 | 10.05862 | 39.31825 | 46.01316 | 29.93675 | 43.92048 | 0.48472 | 0.33143 |
| **Peak17** | 2 | 0 | 606.042 | 1.16064 | 39.15492 | 3.88734 | 292.0404 | 31.20736 | 4.74828 | 0.26486 |
| **Peak18** | 2 | 0 | 615 | 0 | 13.96492 | 13.05117 | 10 | 0 | 0.45587 | 0.42604 |
| **Peak19** | 2 | 0 | 686.2199 | 0.02989 | 20.37281 | 0.1232 | 3038.262 | 19.00747 | 94.94112 | 0.29819 |
| **Peak20** | 2 | 0 | 780 | 0 | 182.1217 | 45.79362 | 497.1027 | 367.544 | 1.73766 | 1.01265 |
| **Peak21** | 2 | 0 | 807 | 0 | 112.0252 | 37.77542 | 511.9411 | 326.4199 | 2.90927 | 0.91297 |
| **Peak22** | 2 | 0 | 841.6948 | 1.58615 | 10 | 0 | 19.59442 | 7.03581 | 1.24742 | 0.44791 |
| **Peak23** | 2 | 0 | 867.8229 | 0.18897 | 11.91111 | 0.73272 | 285.9153 | 19.60981 | 15.28147 | 0.43146 |
| **Peak24** | 2 | 0 | 878.6385 | 0.19395 | 10 | 0 | 207.7201 | 8.40395 | 13.22387 | 0.53501 |
| **Peak25** | 2 | 0 | 902.1378 | 0.132 | 15 | 0 | 549.6089 | 8.47681 | 23.32612 | 0.35977 |
| **Peak26** | 2 | 0 | 913.9509 | 0.13863 | 10 | 0 | 255.6395 | 6.65613 | 16.27452 | 0.42374 |
| **Peak27** | 2 | 0 | 945.8617 | 0.12097 | 8.75046 | 0.3895 | 203.9488 | 7.38589 | 14.83783 | 0.41226 |
